# “Unfocus” on *foc.us*: commercial tDCS headset impairs working memory

**DOI:** 10.1007/s00221-015-4391-9

**Published:** 2015-08-18

**Authors:** Laura Steenbergen, Roberta Sellaro, Bernhard Hommel, Ulman Lindenberger, Simone Kühn, Lorenza S. Colzato

**Affiliations:** 1 Cognitive Psychology Unit, Institute for Psychological Research, Leiden Institute for Brain and Cognition, Leiden University, 2333 AK Leiden, The Netherlands; 2 Max Planck Institute for Human Development, Berlin, Germany

**Keywords:** Working memory, foc.us, tDCS, Updating, *N*-back

## Abstract

In this study, we tested whether the commercial transcranial direct current stimulation (tDCS) headset *foc.us* improves cognitive performance, as advertised in the media. A single-blind, sham-controlled, within-subject design was used to assess the effect of online and off-line *foc.us* tDCS—applied over the prefrontal cortex in healthy young volunteers (*n* = 24) on working memory (WM) updating and monitoring. WM updating and monitoring, as assessed by means of the *N*-back task, is a cognitive-control process that has been shown to benefit from interventions with CE-certified tDCS devices. For both online and off-line stimulation protocols, results showed that active stimulation with *foc.us*, compared to sham stimulation, significantly decreased accuracy performance in a well-established task tapping WM updating and monitoring. These results provide evidence for the important role of the scientific community in validating and testing far-reaching claims made by the brain training industry.

## Introduction

A recent initiative supported by several eminent research institutes and scientists calls for a more critical and active role of the scientific community in evaluating the sometimes far-reaching, sweeping claims from the brain training industry with regard to the impact of their products on cognitive performance (Max Planck Institute on Human Development, Stanford Center on Longevity, 2014). Following this prominent suggestion, we tested whether and to what degree the commercial transcranial direct current stimulation (tDCS) headset *foc.us* improves cognitive performance, as advertised in the media.

tDCS is a noninvasive brain stimulation technique that involves passing a constant direct electrical current through the cerebral cortex (via electrodes placed upon the scalp) flowing from the positively charged anode to the negatively charged cathode (Paulus 2011; Nitsche and Paulus 2011). By doing so, spontaneous cortical excitability is either enhanced or reduced depending on the current polarity: Anodal stimulation leads to a resting-membrane depolarization in the cortical region under the electrode, thus increasing the probability of neural firing, whereas cathodal stimulation leads to a resting-membrane hyperpolarization, thus reducing the probability of neural firing (Nitsche and Paulus 2000; Nitsche et al. 2003). This technique has developed into a promising tool to boost human cognition (Fregni et al. 2005; Fox 2011; Kuo and Nitsche 2012, 2015). Previous studies using tDCS CE-certified devices have shown that excitability-enhancing anodal tDCS applied over the left dorsolateral prefrontal cortex promotes working memory (WM) updating in healthy individuals and patients (for recent reviews, see Brunoni and Vanderhasselt 2014; Kuo and Nitsche 2015), both when combined with excitability-diminishing cathodal tDCS over the right prefrontal cortex, either the right supraorbital region (e.g., Fregni et al. 2005; Boggio et al. 2006; Ohn et al. 2008; Jo et al. 2009; Keeser et al. 2011; Teo et al. 2011) or the right dorsolateral prefrontal cortex (e.g., Oliveira et al. 2013), and when combined with a contralateral extracephalic return electrode (Seo et al. 2011; Zaehle et al. 2011). Such improvements were observed under both online (i.e., stimulation overlapping with the critical task; e.g., Fregni et al. 2005; Ohn et al. 2008; Teo et al. 2011) and off-line (e.g., Ohn et al. 2008; Zaehle et al. 2011; Keeser et al. 2011; Oliveira et al. 2013) stimulation. The ability to monitor and update information in the WM is considered a key cognitive-control function (Miyake et al. 2000) that strongly relies on prefrontal cortex functioning (Curtis and D’Esposito, 2003). Interestingly, WM performance can also be enhanced by video game playing (Colzato et al. 2013a), an activity for which the use of the tDCS headset *foc.us* is recommended to boost performance via (left anodal–right cathodal) prefrontal cortex stimulation.

The aim of the current study was to investigate whether the commercial tDCS headset *foc.us* does in fact improve cognitive performance, as advertised in the media. Given the link between prefrontal cortex activity and WM and the aforementioned studies proving evidence that enhancing left prefrontal cortex activation by means of CE-certified tDCS devices can boost WM performance, we tested whether comparable enhancing effects can be obtained with the commercial tDCS headset *foc.us*. Consistent with previous studies assessing tDCS-induced effects on WM performance (Fregni et al. 2005; Ohn et al. 2008; Jo et al. 2009; Seo et al. 2011; Zaehle et al. 2011; Teo et al. 2011, Keeser et al. 2011; Oliveira et al. 2013), WM updating was assessed by means of the well-established *N*-back task (for a review, see Kane et al. 2007).

In this task, participants are to decide whether each stimulus in a sequence matches the one that appeared n items ago—a task that requires online monitoring, updating, and manipulation of remembered information (Kane et al. 2007). The task gets more difficult as n increases, since this requires more online monitoring, updating, and manipulation of remembered information. We used two conditions: In the 2-back condition, each stimulus was to be compared with the one presented two trials before. In the 4-back condition, each stimulus was to be compared with the one presented four trials before, which implies a higher memory load and greater demands on control resources. In contrast with previous studies, we preferred to include a more challenging 4-back condition instead of the 3-back condition (Teo et al. 2011; Fregni et al. 2005; Ohn et al. 2008), in order to increase the chance to detect possible WM improvements following active *foc.us* tDCS, thereby minimizing potential ceiling effects (cf. Teo et al. 2011; Kuo and Nitsche 2015).

To the degree that the *foc.us* device is comparable to traditional tDCS, we expected participants to be more accurate in monitoring and updating WM when receiving active *foc.us* tDCS than when receiving sham stimulation.

## Experimental procedures

### Participants

The sample size was calculated on the basis of previous studies investigating the effect of tDCS on WM (Fregni et al. 2005; Ohn et al. 2008). Twenty-four undergraduate students of Leiden University (20 females and four males, mean age = 19.6 years, range 18–26) participated in the experiment. Participants were recruited via an online recruiting system and offered course credits for participating in a study on the effects of brain stimulation on memory. Once recruited, participants were randomly assigned to one of the two following experimental groups: off-line stimulation (*N* = 12; two males; mean age = 20.1, SD = 2.5) and online stimulation (*N* = 12; two males; mean age = 19.7, SD = 2.3). Groups did not differ in terms of age, *F* < 1, or gender, *χ* ^2^ = .00, *p* = 1.00. All participants were naïve to *foc.us* tDCS. Participants were screened individually via a phone interview by the same laboratory assistant using the Mini International Neuropsychiatric Interview (MINI). The MINI is a short, structured interview of about 15 min that screens for several psychiatric disorders and drug use, often used in clinical and pharmacological research (Sheehan et al. 1998; Colzato and Hommel 2008; Colzato et al. 2009). Participants were considered suitable to participate in this study if they fulfilled the following criteria: (1) age between 18 and 32 years; (2) no history of neurological or psychiatric disorders; (3) no history of substance abuse or dependence; (4) no history of brain surgery, tumor, or intracranial metal implantation; (5) no chronic or acute medications; (6) no pregnancy; (7) no susceptibility to seizures or migraine; and (8) no pacemaker or other implanted devices.

Prior to the first testing session, all participants received a verbal and written explanation of the *foc.us* tDCS procedure and gave their written informed consent to participate in the study. No information was provided about the different types of stimulation (active vs. sham). The study conformed to the ethical standards of the declaration of Helsinki, and the protocol was approved by the local ethical committee (Leiden University, Institute for Psychological Research).

### Apparatus and procedure

**Fig. 1:**
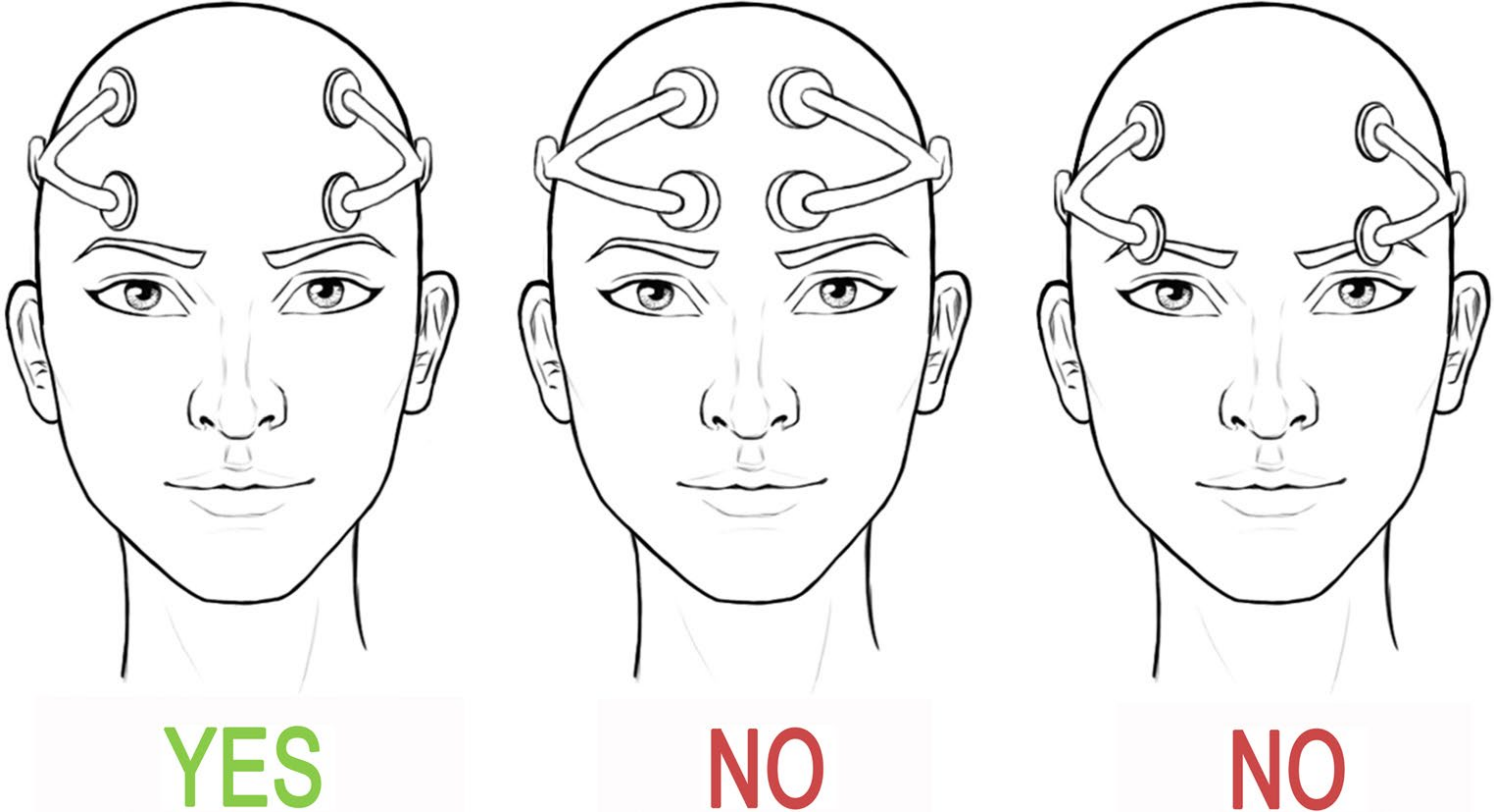
Positioning of the *foc.us* headset on the head as provided by the manufacturer. The correct positioning of *foc.us* is the one displayed in the leftmost panel. Note that this is the only possible allowable montage with this device. Figure designed by the authors

A single-blinded, sham-controlled, randomized crossover within-subject design with counterbalancing of the order of conditions was used to assess the effect of off-line and online *foc.us* tDCS on WM updating in healthy young volunteers. The *foc.us* headset (version 1) was applied over the prefrontal cortex (PFC) according to the manufacturer’s guidelines (see Fig. 1). All participants took part in two sessions (active vs. sham) and were tested individually.

Upon arrival, participants read and signed the informed consent. In the off-line stimulation group, active or sham stimulation was applied for 20 min while at rest. Immediately thereafter, participants were asked to perform the *N*-back task (see Kane et al. 2007, for a review), which lasted for 15 min. In the online stimulation group, participants performed the *N*-back task five minutes after the onset of the stimulation, which was applied throughout the whole task.

At the end of each session, participants were asked to complete a *foc.us* (tDCS) adverse effects questionnaire requiring them to rate, on a five-point (1–5) scale, how much they experienced: (1) headache, (2) neck pain, (3) nausea, (4) muscles contraction in face and/or neck, (5) stinging sensation under the electrodes, (6) burning sensation under the electrodes, (7) uncomfortable (generic) feelings, and (8) other sensations and/or adverse effects. After completion of the second session, participants were debriefed and compensated for their participation.

### Foc.us tDCS commercial device

Direct current was induced by four circular saline-soaked surface sponge electrodes (2.0 cm diameter) and delivered by a *foc.us* tDCS commercial device v1 (http://www.foc.us/; ©FOC.US LABS/EUROPEAN ENGINEERS), a device complying with Part 15 of the Federal Communications Commission (FCC) Rules, but without being CE (European Conformity)-certified. The Federal Code of Regulation (CFR) FCC Part 15 is a common testing standard for most electronic equipment. FCC Part 15 covers the regulations under which an intentional, unintentional, or incidental radiator may be operated without an individual license. FCC Part 15 also covers technical specifications, administrative requirements, and other conditions relating to the marketing of FCC Part 15 devices. Depending on the type of the equipment, verification, declaration of conformity, or certification is the process for FCC Part 15 compliance.

*Foc.us* tDCS was applied on participants’ head according to the instructions provided by the manufacturer, which allow for a single type of electrodes montage, that is, a bipolar-balanced montage (see Nasseri et al. 2015, for a tDCS electrodes montage classification), with anodal stimulation applied over the left prefrontal cortex and cathodal stimulation applied over the right prefrontal cortex (see Fig. 1, leftmost panel). For the active stimulation, a constant current of 1.5 mA was delivered for 20 min with a linear fade-in/fade-out of 15 s. These parameters are within safety limits established from prior work in humans (Nitsche and Paulus 2000; Nitsche et al. 2003, 2004; Poreisz et al. 2007). For sham stimulation, the position of the electrodes, current intensity, and fad-in/fade-out were the same as in the active tDCS, but stimulation was automatically turned off after 30 s, without the participants’ awareness. Hence, participants felt the initial short-lasting skin sensation (i.e., itching and/or tingling) associated with tDCS without receiving any active current for the rest of the stimulation period. Stimulation for 30 s does not induce after effects (Nitsche and Paulus 2000). This procedure has been shown to be effective in blinding participants to the received stimulation condition (see Poreisz et al. 2007; Gandiga et al. 2006; Palm et al. 2013). Consistently, none of the participants was able to determine whether or not he/she received real or sham stimulation. The condition (active vs. sham) and duration of stimulation were controlled by the *foc.us* app iOS (version 2.0) using iPad 4.

### *N*-back task

The experiment was controlled by an ACPI uniprocessor PC running on an Intel Celeron 2.8 gHz processor, attached to a Philips 109B6 17 inch monitor (LightFrame 3, 96 dpi with a refresh rate of 120 Hz). Responses were made by using a QWERTY computer keyboard. Stimulus presentation and data collection were controlled using E-Prime 2.0 software system (Psychology Software Tools, Inc., Pittsburgh, PA).

The two conditions of the *N*-back task were adapted from Colzato et al. (2013a, b). A stream of single visual letters (taken from B, C, D, G, P, T, F, N, L) was presented (stimulus–onset asynchrony 2000 ms; duration of presentation 1000 ms). Participants responded to targets and to nontargets.

Half of the participants pressed the “*z*” key in response to a target and the “*m*” key in response to a nontarget; the other half of the participants received the opposite mapping. Target definition differed with respect to the experimental condition. In the 2-back condition, targets were defined as stimuli within the sequence that were identical to the one that was presented two trials before. In the 4-back condition, participants had to respond if the presented letter matched the one that was presented four trials before. Each condition consisted of a practice block followed by two experimental blocks. The 2-back condition comprised of 106 trials in total (42 target stimuli and 64 nontarget stimuli), whereas the 4-back condition consisted of 110 trials (42 target stimuli and 68 nontarget stimuli). All participants performed the 2-back condition first and then the 4-back condition.

### Statistical analyses

Repeated-measures analyses of variance (ANOVAs) including stimulation protocol (off-line vs. online) as between-subjects factor and condition (Active vs. Sham) as within-subjects factors were performed to compare participants’ self-reports of discomfort about headache, neck pain, nausea, muscles contraction in face and/or neck, stinging sensation under the electrodes, burning sensation under the electrodes, and other uncomfortable (generic) feelings.

For the *N*-back task, practice blocks and either the first two trials (in the 2-back condition) or the first four trials (in the 4-back condition) of each block were excluded from the analyses. Repeated-measures ANOVAs with load (2-back vs. 4-back) and condition (Active vs. Sham) as within-subjects factors and stimulation protocol (off-line vs. online) as between-subjects factor were carried out on reaction times (RTs) on correct trials, as well as for hits, correct rejections, false alarms, and misses in percent. Furthermore, the sensitivity index dˈ was calculated for both active and sham stimulation and the two WM loads separately (see. Haatveit et al. 2010; Buckert et al. 2012). This index, which derives from signal detection theory (Swets, Tanner and Birdsall, 1961), provides a combined measure of correct hits and false alarms and thus reflects participants’ ability to discriminate target from nontargets, with higher dˈ indicating better signal detection. dˈ was computed from hit rate and false alarm (FA) rate using the following formula: Z_HIT_–Z_FA_, where Z represents the z-scores of the two rates (Macmillan and Creelman 1991). The Z transformation was done using the inverse cumulative distribution function in Microsoft Excel 2010 (NORMSINV). Perfect scores were adjusted using these formulas: 1−1/(2*n*) for perfect (i.e., 100 %) hits and 1/(2*n*) for zero false alarms, where *n* was number of total hits or false alarms (Macmillan and Creelman 1991). A significance level of *p* < 0.05 was adopted for all statistical tests.

In addition to standard statistical methods, we calculated Bayesian probabilities associated with the occurrence of the null (p(H_0_|D)) and alternative (p(H_1_|D)) hypotheses, given the observed data (see Masson 2011; Wagenmakers 2007). This method allows making inferences about both significant and nonsignificant effects by providing the exact probability of their occurrence. The probabilities range from with 0 (i.e., no evidence) to 1 (i.e., very strong evidence; see Raftery 1995).

## Results

### Foc.us (tDCS) adverse effects

ANOVAs performed on participants’ self-reports of discomfort revealed significant main effects of condition on self-reports of stinging sensation under the electrode, *F*(1,22) = 10.56, *p* = .004, MSE = 1.044, *η*_*p*_^2^ = 0.32, burning sensation under the electrode, *F*(1,22) = 5.11, *p* = .034, MSE = .587, *η*_*p*_^2^ = 0.19, and other uncomfortable (generic) feelings, *F*(1,22) = 4.64, *p* = .04, MSE = .544, *η*_*p*_^2^ = 0.17, with participants reporting higher discomfort in the active (3.4, 3.0 and 1.9) than in the sham (2.5, 2.5 and 1.4) condition. Finally, a significant interaction involving the factors condition and stimulation protocol was observed on self-reports of headache, *F*(1,22) = 4.24, *p* = .05, MSE = .314, *η*_*p*_^2^ = 0.16. Newman–Keuls post hoc analyses showed that for the off-line stimulation, participants reported higher discomfort in the active than in the sham condition (2.0 vs. 1.4, *p* = .02), whereas no difference between active and sham conditions was observed for participants who received the stimulation during the task (online stimulation; 1.4 vs. 1.3, *p* = .72). No other significant source of variance was observed, *F*_*s*_ ≤ 3.12, *p*_*s*_ ≥ .09.

### *N*-back task

Table 1 shows mean RTs (in milliseconds: ms), hits, correct rejections, false alarms, and misses (in percent) for the *N*-back task separately for off-line and online stimulations and for active and sham conditions.

**Table 1:** Mean RTs (in ms), hits, correct rejections, false alarms, and misses (in percent) for the *N*-back task as a function of condition (sham vs. active) and stimulation protocol (off-line vs. online stimulation)

*N*-back (WM monitoring/updating)	Off-line stimulation		Online stimulation	
	Sham	Active	Sham	Active
2-back				
Reaction times (ms)	480 (19.1)	487 (16.5)	505 (19.1)	496 (16.5)
Hits (%)	90.9 (2.0)	88.5 (2.2)	90.7 (2.0)	85.5 (2.2)
Correct rejections (%)	93.1 (2.8)	92.9 (1.7)	92.1 (2.8)	91.1 (1.7)
False alarms (%)	6.9 (2.8)	7.1 (1.7)	7.9 (2.8)	8.9 (1.7)
Misses (%)	9.1 (2.0)	11.5 (2.2)	9.3 (2.0)	14.5 (2.2)
4-back				
Reaction times (ms)	561 (11.6)	575 (15.7)	575 (11.6)	559 (15.7)
Hits (%)	63.3 (3.7)	59.9 (2.9)	68.7 (3.7)	64.1 (2.9)
Correct rejections (%)	78.5 (3.2)	82.1 (2.3)	78.8 (3.2)	79.0 (2.3)
False alarms (%)	21.5 (3.2)	17.9 (2.3)	21.2 (3.2)	21.0 (2.3)
Misses (%)	36.7 (3.7)	40.1 (2.9)	31.3 (3.7)	35.9 (2.9)

Standard errors are shown within parentheses

**Fig. 2:**
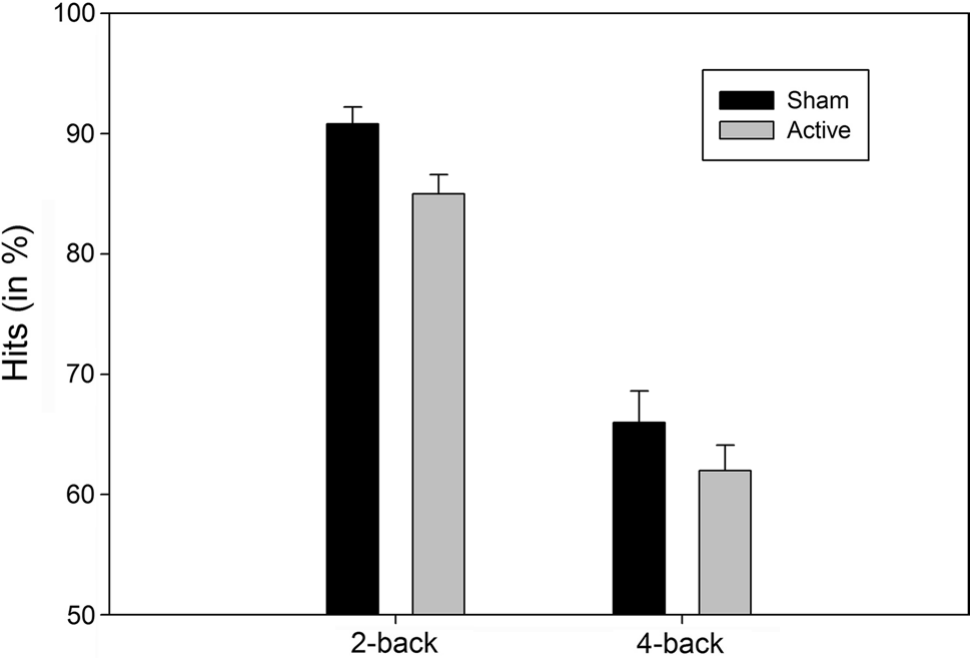
Mean hits (in  %) as a function of load (2-back vs. 4-back) and condition: active and sham. *Vertical capped lines* *atop* *bars* indicate standard error of the mean

Load (i.e., 2-back vs. 4-back) affected all dependent measures, showing that higher load increased RTs (568 vs. 492 ms), *F*(1,22) = 63.80, *p* = .0001, MSE = 2148.196, *η*_*p*_^2^ = 0.74, p(H_1_|D) > .99, and reduced hit rates (89 vs. 64 %), *F*(1,22) = 125.60, *p* = .0001, MSE = .012, *η*_*p*_^2^ = 0.85, p(H_1_|D) > .99. Higher load also produced fewer correct rejections (92 vs. 80 %), but more false alarms (8 vs 20 %), *F*(1,22) = 38.34, *p* = .0001, MSE = .010, *η*_*p*_^2^ = 0.64, p(H_1_|D) > .99, and misses (11 vs. 36 %), *F*(1,22) = 125.60, *p* = .0001, MSE = .012, *η*_*p*_^2^ = 0.85, p(H_1_|D) > .99, than the lower load did. Most importantly, with regard to the effect of condition, active stimulation, as compared to sham, significantly reduced hits (75 vs. 78 %) and increased misses (26 vs. 22 %), *F*(1,22) = 5.62, *p* = .027, MSE = .006, *η*_*p*_^2^ = 0.20, p(H_1_|D) = .76, but it did not affect RTs, false alarms, correct rejections, *F* < 1, *p* ≥ .71, p(H_0_|D) ≥ .81, [d′_(sham)_ = 2.2, d′_(active)_ = 2.0] (see Fig. 2). No further significant source of variance was observed, *F*_*s*_ ≤ 2.5, *p*_*s*_ ≥ .13, *p*_*s*_(H_0_|D) ≥ .60.

## Discussion

The present study is the first to demonstrate that prefrontal cortex stimulation delivered using the commercial *foc.us* tDCS headset (version 1) impairs the ability to monitor and update information in the WM. Results showed that regardless of the adopted protocol (online or off-line stimulation), active stimulation with *foc.us* significantly decreased hits and increased misses in a WM monitoring task compared to sham stimulation. Given that WM updating is a key cognitive-control function (Miyake et al. 2000), the present findings do not support the claims that the use of *foc.us* tDCS (version 1) headset can improve cognitive performance. Instead, our results suggest that the use of this device can actually be detrimental and, as such, cannot be regarded as an alternative to CE-certified tDCS devices, the use of which has been demonstrated to be successful in promoting WM (Fregni et al. 2005; Kuo and Nitsche 2012; Boggio et al. 2006; Ohn et al. 2008; Jo et al. 2009; Teo et al. 2011; Seo et al. 2011; Zaehle et al. 2011). In contrast to such devices, the *foc.us* device is not CE-certified but complies only with Part 15 of the FCC Rules.

Given that, as advertised in the media, the use of *foc.us* is quite popular among young people to improve their gaming performance, future research will need to explore the effects of prolonged use of *foc.us* on the brain. Moreover, given that tDCS has the potential to induce significant alterations of functional connectivity (e.g., Polanía et al. 2011; Keeser et al. 2011), follow-up studies should assess whether the use of *foc.us* produces prefrontal functional connectivity changes and how these possible changes relate to behavioral performance decrements.

From a more general point of view, *foc.us* is just one example of a device that can easily be purchased and, without any control or expert knowledge, used by anyone. The results of our study are straightforward in showing that the claims made by companies manufacturing such devices need to be validated. To conclude, even if the consequences of long-term or frequent use of the *foc.us* device are yet to be demonstrated, our findings provide strong support for the claim that the scientific community should play a more critical and active role in validating and testing far-reaching claims made by the brain training industry.
